# Indole-3-carboxaldehyde from *Limosilactobacillus reuteri* targets the DUSP1/ERK/NOX2/ROS axis to enhance the bactericidal activity of macrophages and protects against sepsis

**DOI:** 10.1080/19490976.2026.2671382

**Published:** 2026-05-14

**Authors:** Zhiwang Li, Peiyu Li, Tian Peng, Xing Zhou, Yihong Liu, Chenmu Ai, Neng Xiao, Shunxin Song, Xiaomei Lei, Junyong Liu, Wenlei Wang, Pan Zhou, Zhangyong Li, Zhanhong Liu, Xingui Dai, Zhiming Zhang, Tao Li

**Affiliations:** a Department of Anesthesiology, The First People’s Hospital of Chenzhou, Medical Education Center of Jinan University, Chenzhou, China; b Department of Critical Care Medicine, The First People’s Hospital of Chenzhou, The First School of Clinical Medicine, Southern Medical University, Chenzhou, China; c The First Affiliated Hospital of Jinan University, Guangzhou, China; d Department of Gastroenterology, The First People’s Hospital of Chenzhou, Chenzhou, China; e The First People's Hospital of Chenzhou, The First Clinical College, Xiangnan University, Chenzhou, China; f Department of Critical Care Medicine, The First People’s Hospital of Chenzhou, The Affiliated Chenzhou Hospital, Hengyang Medical School, University of South China, Chenzhou, China; g Department of Pathophysiology, Guangdong Provincial Key Laboratory of Cardiac Function and Microcirculation, School of Basic Medical Sciences, Southern Medical University, Guangzhou, China; h Department of Critical Care Medicine,Nanfang Hospital, Southern Medical University, Guangzhou, China; i Department of Pathophysiology, Guangdong Provincial Key Laboratory of Proteomics, School of Basic Medical Sciences, Southern Medical University, Guangzhou, China; j Department of Emergency Medicine, The First People’s Hospital of Chenzhou, Hunan University of Chinese Medicine, Chenzhou, China

**Keywords:** Sepsis, *Limosilactobacillus reuteri*, indole-3-carboxaldehyde, DUSP1, macrophage, phagocytosis, ROS

## Abstract

The gut microbiota plays a critical regulatory role in the pathogenesis of sepsis, yet the immunomodulatory mechanisms of *Limosilactobacillus reuteri* (*L. reuteri*) and its metabolites in sepsis remain to be fully elucidated. This study found that the abundance of intestinal *L. reuteri* was significantly reduced in patients with bacterial sepsis and showed a negative correlation with disease severity. In a mouse model of sepsis induced by cecal ligation and puncture, fecal microbiota transplantation and live bacterial supplementation further confirmed that live *L. reuteri* effectively attenuated sepsis progression. Integrated metabolomic and network pharmacological analysis identified indole-3-carboxaldehyde (IAld), a metabolite derived from *L. reuteri*, which enhances macrophage bactericidal function and alleviates sepsis-associated organ damage. Mechanistically, IAld directly targets DUSP1 in macrophages, inhibits its phosphatase activity, thereby promoting ERK phosphorylation, upregulating NOX2 expression, stimulating reactive oxygen species production, and ultimately enhancing bacterial clearance. Notably, circulating IAld levels in septic patients were significantly inversely correlated with SOFA score, APACHE II score, and arterial lactate levels, and IAld safely enhanced the bactericidal function of human macrophages in vitro. This study is the first to systematically demonstrate that *L. reuteri* and its metabolite IAld exert a protective effect against sepsis through the DUSP1/ERK/NOX2/ROS axis, providing novel mechanistic insights and potential therapeutic targets for immunometabolic intervention in sepsis.

## Introduction

Sepsis is a life-threatening organ dysfunction caused by a dysregulated host response to infection.^1^ ^,^ ^2^ Despite advances in diagnosis and management, mortality remains high—approximately 25–30% overall, rising to 40–50% in septic shock—posing a critical global health challenge.^3-5^ Uncontrolled infection, triggering excessive inflammatory cascades, is a pivotal driver of sepsis.^6^ Consequently, timely clearance of pathogens is critical for preventing the progression of sepsis.^7^ While antibiotics remain a cornerstone of sepsis treatment, broad-spectrum regimens can exacerbate intestinal dysbiosis and increase the risk of sepsis recurrence.^8^ ^,^ ^9^ Furthermore, antibiotic misuse can induce or exacerbate organ dysfunction through mechanisms such as mitochondrial toxicity, immune cell dysfunction, and direct organ injury,^10^ and promote the emergence of multidrug-resistant organisms.^11^ ^,^ ^12^ Therefore, careful risk-benefit assessment of antibiotic use is essential in sepsis management. Given these limitations, enhancing intrinsic host defense mechanisms to improve pathogen clearance has emerged as a promising therapeutic avenue.^13^

The intestine is considered to play a pivotal role in sepsis-induced multiple organ dysfunction syndrome.^14^ ^,^ ^15^ Among the mechanisms, gut microbiota dysbiosis can exacerbate inflammatory responses through metabolic and immune regulatory pathways, thereby contributing to the pathogenesis and progression of sepsis;^16-19^ furthermore, depletion of the gut microbiota has been shown to impair the pathogen-clearing capacity of alveolar macrophages and Kupffer cells.^20-23^ As uncontrolled infection is a critical step in initiating excessive inflammation, targeting the gut microbiome represents a potential therapeutic strategy. A recent study revealed that phenylacetic acid, produced by the intestinal fungus *Candida albicans*, enhances macrophage bacterial phagocytosis.^13^ However, the opportunistic nature of *C. albicans* poses significant safety concerns for clinical translation. Another study reported that the commensal *Parabacteroides goldsteinii* exhibits antibacterial effects in aged male mice, though its efficacy in broader populations requires further evaluation.^24^ Thus, identifying safe probiotic strains capable of enhancing macrophage bactericidal function is urgently needed to inform novel sepsis prevention and treatment strategies.

In this study, we employed a cecal ligation and puncture (CLP)-induced murine sepsis model combined with a clinical septic patient cohort. Using 16S rDNA sequencing and untargeted metabolomics, we identified the gut commensal bacterium *Limosilactobacillus reuteri (L. reuteri)* and its metabolite indole-3-carboxaldehyde (IAld) as contributors to beneficial outcomes in sepsis. Through subsequent *in vivo* and in vitro experiments, we systematically evaluated the protective roles and underlying mechanisms of *L. reuteri* and IAld. Mechanistically, IAld directly targets DUSP1 in macrophages, inhibits its phosphatase activity, thereby promoting ERK phosphorylation, upregulating NOX2 expression, stimulating reactive oxygen species production, and ultimately enhancing bacterial clearance. Our work demonstrates for the first time that *L. reuteri* alleviates sepsis by promoting macrophage-mediated pathogen clearance via its metabolite indole-3-carboxaldehyde (IAld), thereby providing a novel therapeutic strategy and experimental foundation for sepsis management.

## Results

### Intestinal *L. reuteri* is associated with sepsis progression in humans and mice

**Figure 1.**
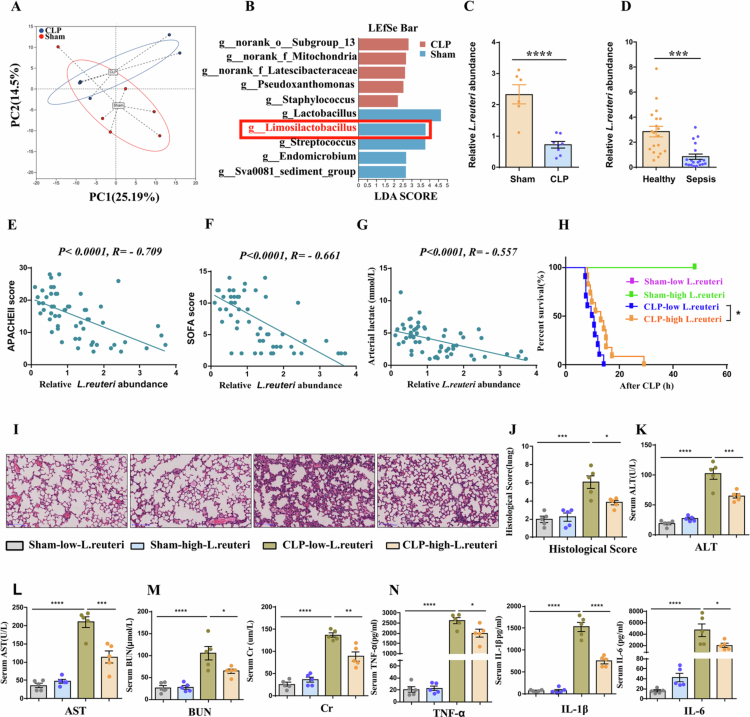
Intestinal *L. reuteri* is associated with sepsis progression in humans and mice. (A) Principal component analysis (PCA) of gut microbiota from sham and septic (CLP) mice (*n* = 6 per group) based on weighted Unifrac distance. Ellipses represent 95% confidence intervals. (B) Linear discriminant analysis (LDA) effect size (LEfSe) analysis identifying differentially abundant bacterial taxa between sham and CLP groups (LDA > 3, *p *<* *0.05. *n* = 6 per group). Blue and red indicate taxa enriched in the sham and CLP groups, respectively. (C) Quantitative PCR (qPCR) analysis of fecal *L. reuteri* abundance in sham and CLP mice (*n* = 6–8). (D) qPCR analysis of fecal *L. reuteri* relative abundance in healthy controls and septic patients (*n* = 19 per group). (E–G) Spearman correlation analysis between fecal *L. reuteri* relative abundance and Sequential Acute Physiology and Chronic Health Evaluation II (APACHE II) score (E), Organ Failure Assessment (SOFA) score (F), and arterial lactate levels (G) in septic patients (*n* = 56). (H) Mice receiving high *L. reuteri* abundance feces exhibited significantly prolonged survival time after CLP. (*n* = 5–11, with no mortality in any Sham group, survival curves overlapped completely, showing only a single line for all Sham subgroups). (I–J) Representative hematoxylin and eosin (H&E)-stained lung sections and corresponding histopathological injury scores (scale bar: 100 µm; original magnification: × 100; *n* = 5). (K–L) Plasma ALT and AST levels (*n* = 5). (M) Plasma BUN and creatinine levels (*n* = 5). (N) Plasma concentrations of the inflammatory cytokines TNF-*α*, IL-1β, and IL-6 (*n* = 5). Data are presented as mean ± SEM. Statistical significance was determined by PERMANOVA (A), unpaired two-tailed Student's t test (C, D), one-way ANOVA with Bonferroni’s post hoc tests (J–M), Spearman rank-order correlation test (E–G), and log-rank test (H). *P <* *0.05, **P <* *0.01, ***P <* *0.001, ****P <* *0.0001; ns, not significant.

To investigate the association between gut microbiota and sepsis pathogenesis, we performed 16S rDNA sequencing of fecal samples from sham and cecal ligation and puncture (CLP) mice. Principal component analysis (PCA) revealed a significant separation in gut microbial community structure between CLP and sham groups (Figure 1A). LEfSe analysis further indicated a marked reduction in the genus *Limosilactobacillus* in CLP mice (Figure 1B). Given that specific *Limosilactobacillus* strains exhibit therapeutic potential in various diseases—such as *L. reuteri* alleviating cisplatin-induced ovarian toxicity.^25^ *L. fermentum* SLAM216 improving atopic dermatitis.^26^ and *L. mucosae*-derived vesicles promoting intestinal homeostasis.^27^ —we used qPCR to validate changes in these species. We observed a significant reduction in *L. reuteri* abundance in CLP mice (Figure 1C), but not in *L. fermentum* or *L. mucosae* (Supplemental information Fig. S1A–S1D).

We next enrolled 19 septic patients and 19 matched healthy controls, and found that fecal *L. reuteri* abundance was significantly lower in patients than in healthy controls (Figure 1D, clinical data in Supplemental information Table S1). In an expanded cohort of 56 patients (clinical data in Supplemental information Table S2), *L. reuteri* abundance correlated negatively with APACHE II score, SOFA score, and serum arterial lactate levels (Figure 1E–G). To evaluate the causal relationship between gut microbiota and the disease, we performed fecal microbiota transplantation (FMT) with reference to established protocols.^28^ Specifically, from 56 septic patients, we selected a quarter of fecal samples with either very high or very low *L. reuteri* abundance samples and divided them equally into two groups. We transplanted fecal microbiota from these two groups into recipient mice and performed CLP surgery. Interestingly. Mice receiving feces with high *L. reuteri* abundance showed prolonged survival time after CLP compared to those receiving low *L. reuteri* abundance feces, as confirmed by Kaplan–Meier analysis (Figure 1H). Furthermore, mice in the group receiving feces with high *L. reuteri* abundance demonstrated markedly attenuated histopathological damage in lung tissues (Figure 1I–J), accompanied by significantly reduced plasma levels of hepatic injury markers (ALT, AST) (Figure 1K and L), renal injury markers (BUN, Cr) (Figure 1M), and inflammatory cytokines (TNF-*α*, IL-1β, IL-6) (Figure 1N). Collectively, these results demonstrate that intestinal *L. reuteri* abundance is a critical factor influencing the severity of sepsis.

### Live *L. reuteri* exerts protective effects against sepsis in mice

**Figure 2.**
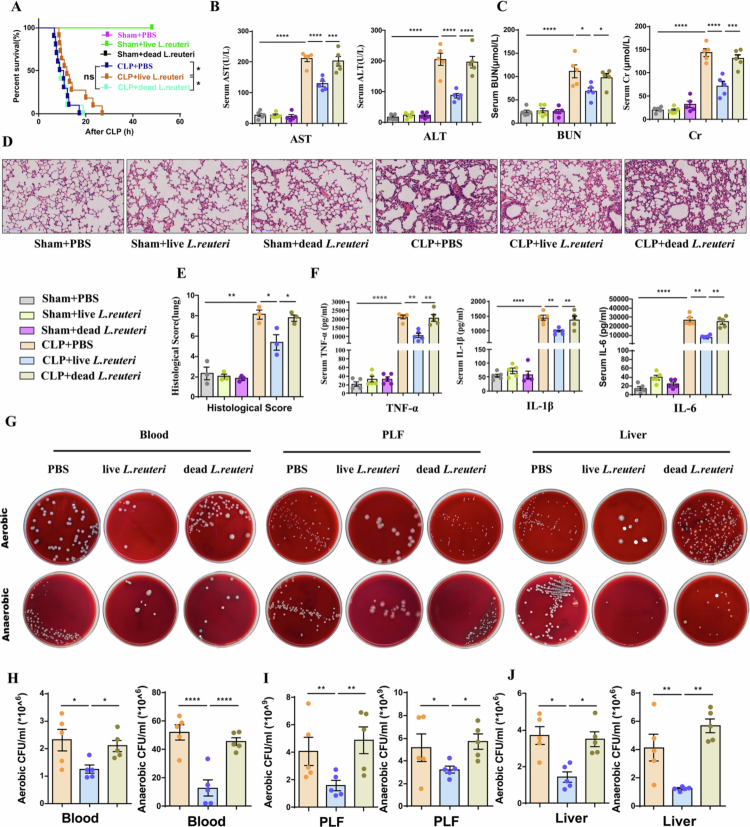
Live *L. reuteri*exerts protective effects against sepsis in mice. Mice were orally gavaged with PBS, live *L. reuteri*, or dead *L. reuteri* (300 µL per dose, 1 × 10⁹ CFU/mL) for three consecutive days, followed by sham or CLP surgery. (A) The survival time of mice pretreated with live *L. reuteri* was prolonged after CLP challenge compared to that of mice pretreated with PBS or dead *L. reuteri*. (*n* = 5–10, with no mortality in any Sham group, survival curves overlapped completely, showing only a single line for all Sham subgroups). (B) Plasma ALT and AST levels (*n* = 5). (C) Plasma BUN and creatinine levels (*n* = 5). (D–E) Representative hematoxylin and eosin (H&E)-stained lung sections and corresponding histopathological injury scores (scale bar: 100 µm; original magnification: × 100; *n* = 3). (F) Plasma concentrations of the inflammatory cytokines TNF-*α*, IL-1β, and IL-6 (*n* = 5). (G) Representative images of bacterial burden in the blood, peritoneal lavage fluid, and liver samples of CLP mice after incubation under aerobic or anaerobic conditions at 37 °C for 14–16 hours. (H–J) Quantitative bacterial counts in blood (H), peritoneal lavage fluid (I), and liver (J) samples from CLP model mice (*n* = 5). Data are presented as mean ± SEM. One-way ANOVA with Bonferroni's post hoc tests except for log-rank test for survival rate analysis (A); *P <* *0.05, **P <* *0.01, ***P <* *0.001, ****P < 0.0001; ns, not significant.

Next, to determine the direct effect of *L. reuteri* on sepsis, we subjected mice to pretreatment with PBS, live *L. reuteri*, or heat-inactivated *L. reuteri* (dead *L. reuteri*) followed by sham or CLP surgery. The survival time of mice pretreated with live *L. reuteri* was prolonged after CLP challenge compared to that of mice pretreated with PBS or dead *L. reuteri*. (Figure 2A). Furthermore, pretreatment with live *L. reuteri* markedly reduced plasma levels of hepatic injury markers (ALT, AST) (Figure 2B) and renal injury markers (BUN, Cr) (Figure 2C), attenuated histopathological damage in lung tissues (Figure 2D and E), and was accompanied by significantly reduced plasma levels of inflammatory cytokines (TNF-*α*, IL-1β, and IL-6) (Figure 2F). Given the critical role of bacterial clearance in sepsis,^29^ we measured bacterial counts in blood, peritoneal lavage fluid (PLF), and liver samples from mice 12 hours after CLP surgery. Live *L. reuteri*-pretreated significantly reduced the bacterial count (Figure 2G–J). These data indicate that live *L. reuter*—but not dead *L. reuteri* attenuates organ injury and prolongs the survival time of septic mice.

### *L.* *reuteri* protects against sepsis via secretion of indole-3-carboxaldehyde (IAld)

**Figure 3.**
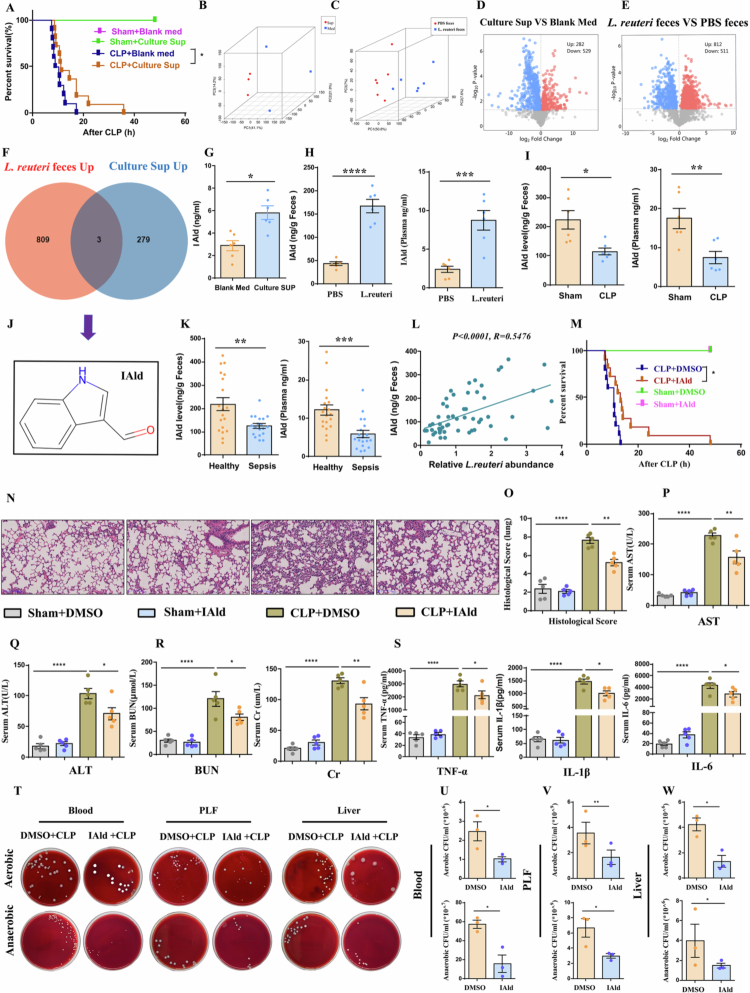
L. reuteri protects against sepsis via secretion of indole-3- carboxaldehyde (IAld). Mice were orally gavaged with blank medium or *L. reuteri* culture supernatant for 5 consecutive days, followed by sham or CLP surgery. (A) Mice receiving *L. reuteri* culture supernatant exhibited significantly prolonged survival time after CLP. (*n* = 6-15, with no mortality in any Sham group, survival curves overlapped completely, showing only a single line for all Sham subgroups). (B) Principal component analysis (PCA) of metabolic profiles from blank medium versus *L. reuteri* culture supernatant (*n* = 3). (C) PCA of fecal metabolites from mice colonized with PBS or live *L. reuteri* (*n* = 6). (D) Volcano plot comparing metabolic profiles of blank medium and *L. reuteri* culture supernatant. Red and blue points represent metabolites significantly enriched or reduced, respectively (log₂FC ≥ 2, *P* < 0.01; *n* = 3). (E) Volcano plot comparing intestinal metabolites from PBS- versus *L. reuteri*-colonized mice (*n* = 6). (F) Venn diagram illustrating metabolites commonly enriched in feces from *L. reuteri*-colonized mice and in *L. reuteri* culture supernatant. (G) IAld concentration in blank medium versus *L. reuteri* culture supernatant (*n* = 6). (H) Fecal and plasma IAld levels in mice colonized with PBS or live *L. reuteri* (*n* = 6). (I) IAld levels in fecal and plasma samples of mice at 12 hours after CLP surgery (*n* = 6). (J) Chemical structure of IAld. (K) IAld levels in feces and plasma from septic patients and healthy controls (*n* = 19). (L) Spearman correlation analysis revealed a significantly positive correlation between the relative abundance of *L. reuteri* in fecal samples and IAld levels in patients with sepsis (*n* = 56). (M) IAld pretreatment significantly prolonged survival time after CLP. (*n* = 5-11, with no mortality in any Sham group, survival curves overlapped completely, showing only a single line for all Sham subgroups.) (N–O) Representative hematoxylin and eosin (H&E)-stained lung sections and corresponding histopathological injury scores (scale bar: 100 µm; original magnification: × 100). (P–Q) Plasma ALT and AST levels (*n* = 5). (R) Plasma BUN and creatinine levels (*n* = 5). (S) Plasma concentrations of the inflammatory cytokines TNF-*α*, IL-1β, and IL-6 (*n* = 5). (T) Representative images of bacterial burden in mouse blood, PLF, and liver samples. (U–W) Quantitative bacterial counts in blood (U), peritoneal lavage fluid (V), and liver (W) samples from CLP model mice (*n* = 3). Data are presented as mean ± SEM; log-rank test (A, M), PERMANOVA for multivariate comparisons (B–C), Spearman's rank-order correlation for correlation analysis (L); unpaired two-tailed Student's t-test (G-J,T-V); one-way ANOVA with Bonferroni’s post hoc tests (O-S); *P <* *0.05, **P <* *0.01, ***P <* *0.001, ****P <* *0.0001; ns, not significant.

Based on the finding that “only live *L. reuteri* exerts protective effects,” we speculated that its beneficial role may be associated with bioactive substances produced by the live bacteria. Building on this, we further hypothesized that the protective effect of *L. reuteri* might similarly depend on metabolites derived from its live form. To test this hypothesis, mice were orally gavaged with blank medium (Med) or *L. reuteri* culture supernatant (Sup) for 5 consecutive days, followed by sham or CLP surgery. The results showed that mice pretreated with *L. reuteri* culture supernatant survived longer than those receiving blank medium after CLP (Figure 3A), indicating that *L. reuteri* culture supernatant confers protection. To delineate the key metabolites responsible for the protective effects of *L. reuteri* against sepsis, we conducted untargeted metabolomic analysis of feces from *L. reuteri*-mono-colonized mice and its culture supernatant. Principal component analysis revealed distinct metabolic profiles that clearly separated *L. reuteri*- associated samples from blank medium in both *in vivo* and in vitro settings (Figure 3B and C). Volcano plot analysis identified metabolites significantly upregulated by *L. reuteri* (Figure 3D and E), and a Venn diagram highlighted three overlapping metabolites common to both datasets (Figure 3F). From these, we selected indole-3-carboxaldehyde (IAld) for further investigation, as it consistently exhibited the highest fold-increase. Targeted metabolomics validated that IAld was enriched in the culture supernatant of *L. reuteri* (Figure 3G). Accordingly, mice colonized with live *L. reuteri* showed significantly elevated levels of IAld in both feces and plasma compared to non-colonized controls (Figure 3H), confirming that *L. reuteri* effectively produces IAld in vitro and *in vivo*.

To investigate the role of IAld in the pathogenesis of sepsis, we induced sepsis using the cecal ligation and puncture (CLP) model and measured its concentrations. Dynamic monitoring revealed that IAld levels in both fecal and plasma samples showed significant decreases at 6 hours (Fig. S1G-H) and 12 hours (Figure 3I) after CLP surgery. Interestingly, mice receiving feces with high *L. reuteri* abundance elevated levels of IAld in both feces and plasma compared to those receiving low *L. reuteri* abundance feces (Fig. S1E-F). Clinically, this finding was mirrored by markedly lower IAld concentrations in feces and plasma from septic patients compared to healthy controls (Figure 3K; clinical data in Supplemental information Table S1). Notably, the abundance of *L. reuteri* was positively correlated with IAld levels (Figure 3L; clinical data in Supplemental information Table S2). Collectively, these results demonstrate that IAld production is impaired during sepsis and suggest its potential as a key regulatory metabolite in the disease pathogenesis.

To achieve effective plasma concentrations, mice were orally administered DMSO or IAld (20 mg/kg) for three consecutive days,^30-32^ and CLP surgery was performed 3 hours after the last administration. Kaplan–Meier analysis revealed that mice receiving IAld pretreatment showed prolonged survival time after CLP compared to those receiving DMSO pretreatment (Figure 3M). Furthermore, mice receiving IAld pretreatment demonstrated markedly attenuated histopathological damage in lung tissues (Figure 3N–O), accompanied by significantly reduced plasma levels of hepatic injury markers (AST and ALT) (Figure 3P–Q), renal injury markers (BUN, Cr)(Figure 3R), and inflammatory cytokines (TNF-*α*, IL-1β, IL-6) (Figure 3S). We also measured bacterial counts 12 h after CLP. IAld pretreatment significantly reduced bacterial counts in blood, peritoneal lavage fluid (PLF), and liver (Figure 3T–W). In summary, these results indicate that IAld alleviates sepsis-induced multiple organ injury and prolongs the survival time of septic mice.

### IAld alleviates sepsis by enhancing the phagocytic and bacterial-killing capacities of macrophages rather than neutrophils

**Figure 4.**
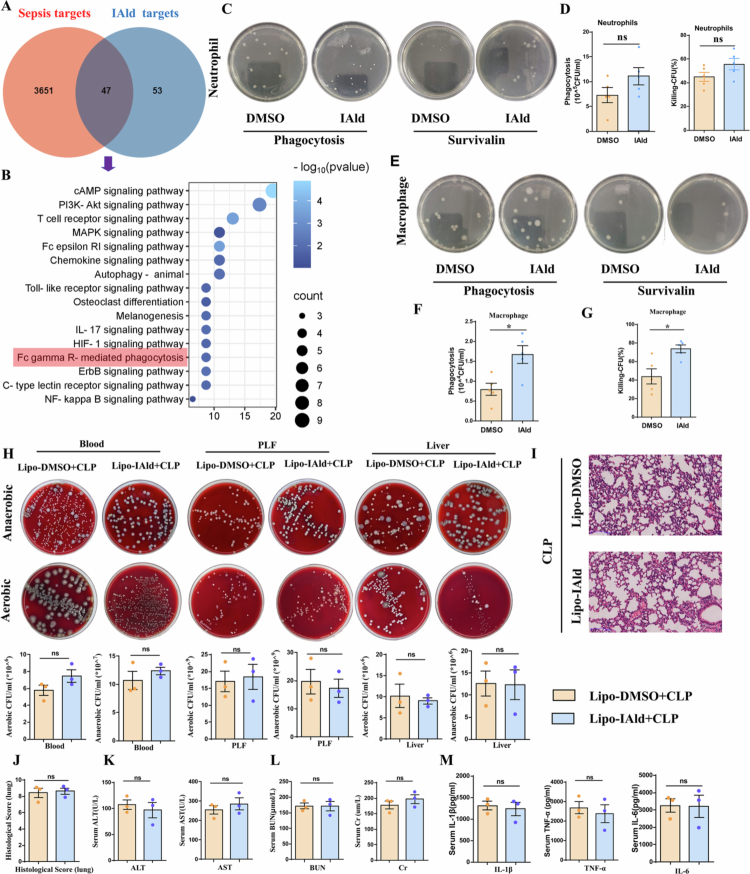
IAld alleviates sepsis by enhancing the phagocytic and bactericidal capacity of macrophages rather than neutrophils. (A) Venn diagram analysis revealed an intersection between the targets of IAld and sepsis-related targets, identifying 47 potential therapeutic targets for IAld against sepsis. (B) KEGG enrichment analysis revealed that some anti-sepsis target genes of IAld were significantly enriched in the Fc gamma R-mediated phagocytosis pathway. (C) Bone marrow-derived neutrophils (BMDNs) were pretreated with IAld for 12 hours and then co-incubated with *E. coli*. Representative images of phagocytic activity and bacterial killing capacity are shown. (D) Quantitative results of phagocytosis and bacterial killing capacity in BMDNs (*n* = 5). (E) BMDMs were pretreated with IAld for 12 hours and then co-incubated with *E. coli*. Representative images of phagocytic activity and bacterial killing capacity are shown. (F) Quantitative results of phagocytosis capacity in BMDMs (*n* = 5). (G) Quantitative results of bacterial killing capacity in BMDMs (*n* = 5). (H) Macrophage-depleted mice were orally pretreated with DMSO or IAld prior to CLP surgery. Representative images and quantitative results of bacterial counts in blood, peritoneal lavage fluid, and liver samples (*n* = 3). (I–J) Representative H&E staining images and corresponding histological scores of lung tissues (scale bar: 100 µm, magnification: × 100; *n* = 3). (K) Plasma ALT and AST levels (*n* = 3). (L) Plasma BUN and Cr levels (*n* = 3). (M) Plasma levels of inflammatory factors IL-1β, TNF-*α*, and IL-6 (*n* = 3). Data are presented as mean ± SEM. Statistical significance was determined by the unpaired two-tailed Student's t-test. *P <* *0.05, **P <* *0.01, ***P <* *0.001, ****P <* *0.0001; ns, not significant.

To identify the therapeutic targets of IAld for sepsis treatment, we initially predicted 3698 potential sepsis-related targets using the GeneCards and OMIM databases. To precisely determine the targets of indole-3-carboxaldehyde (IAld) (a promising therapeutic compound), we conducted searches in PubChem and SwissTargetPrediction databases, identifying 100 potential targets. The intersection between IAld targets and sepsis targets yielded 47 common targets (Figure 4A). Subsequently, we conducted Kyoto Encyclopedia of Genes and Genomes (KEGG) pathway enrichment analysis on these 47 shared target genes to identify key molecular pathways associated with IAld. Interestingly, several genes showed significant enrichment in the Fc gamma R-mediated phagocytosis, which is known to influence bacterial load and clearance in sepsis (Figure 4B). This finding suggests that IAld may protect the host from sepsis by enhancing the phagocytic capacity of immune cells to eliminate bacteria, thereby effectively reducing bacterial burden.

To investigate whether IAld modulates the intrinsic antibacterial functions of phagocytes, we isolated bone marrow-derived neutrophils (BMDNs) and macrophages (BMDMs). Cells were pretreated with IAld or DMSO. Preincubation with IAld did not affect the phagocytic and bacterial-killing capacities of BMDNs against live *E. coli* (Figure 4C and D). In contrast, IAld-treated BMDMs exhibited significantly enhanced phagocytosis of *E. coli* compared to the DMSO-treated control (Figure 4E and F). Furthermore, following bacterial infection, IAld-treated BMDMs also demonstrated markedly stronger bacterial-killing capacity than control cells (Figure 4E and G). These results indicate that the protective effect of IAld is associated specifically with the enhancement of macrophage bactericidal activity, rather than a general effect on all phagocytes. Additionally, we evaluated the potential cytotoxicity of IAld (0–500 μM) on bone marrow‑derived macrophages (BMDMs; Fig. S2A), neutrophils (BMDNs; Fig. S2B), and dendritic cells (DCs; Fig. S2C) using the Cell Counting Kit-8 (CCK-8) assay. Results demonstrated that none of the tested concentrations caused a significant reduction in cell viability. Furthermore, immunofluorescence staining revealed that IAld had no significant effect on the activation status of dendritic cells in the liver, lungs, or kidneys of CLP model mice (Fig. S2E-G).

To determine whether macrophages are essential for the protective effects of IAld against sepsis, we depleted macrophages in mice by pretreating them with clodronate liposomes (Lipo) prior to CLP surgery. Macrophage depletion abolished the protective effects of IAld, as evidenced by significantly impaired bacterial clearance (Figure 4H) in septic mice and attenuated organ protection (Figure 4I-4L). Consistent with this loss of protection, IAld no longer suppressed the elevation of plasma inflammatory cytokines in macrophage-depleted mice (Figure 4M). Notably, mice were treated with an anti-Ly6G antibody to deplete neutrophils prior to CLP. After neutrophil depletion, IAld-treated septic mice still exhibited enhanced organ protection (Fig. S3A-D), improved bacterial clearance (Fig. S3E-G), and significantly reduced expression of inflammatory factors (Fig. S3H-J). Collectively, these findings demonstrate that macrophages are required for IAld-mediated protection in sepsis.

### IAld enhances the bacterial-killing capacity of macrophage through the ERK/NOX2 pathway-mediated ROS generation

**Figure 5.**
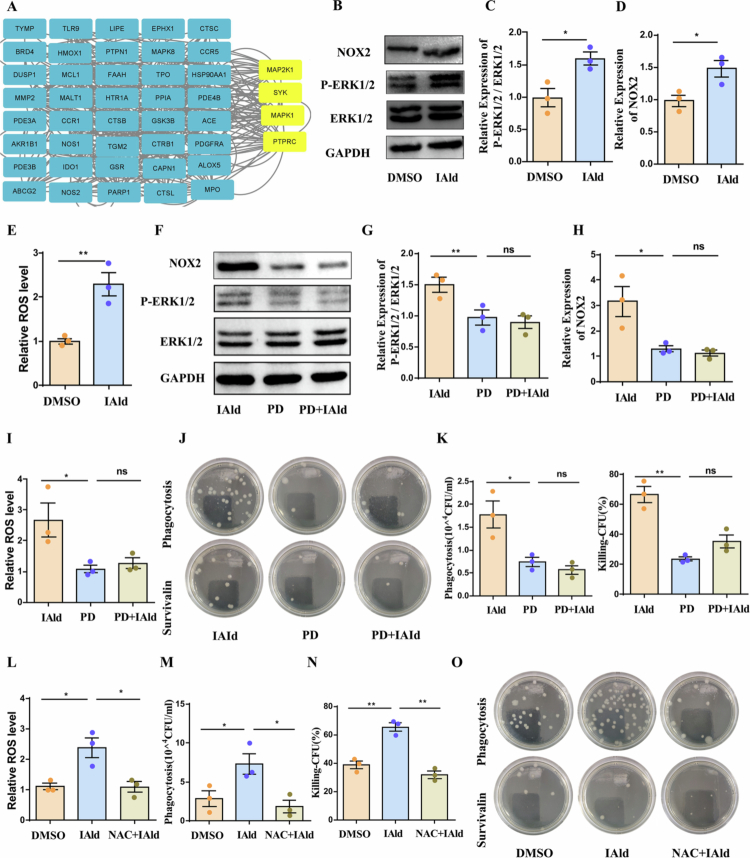
IAld enhances macrophage bactericidal capacity through the ERK/NOX2 pathway-mediated ROS generation. (A) Protein interaction network of the 47 predicted targets. (B–D) Representative Western blot and quantification of ERK1/2, *p*-ERK1/2, and NOX2 expression in BMDMs treated with DMSO or IAld (*n* = 3). (E) IAld significantly increased ROS levels in BMDMs (*n* = 3). (F–H) Western blot and quantification of ERK1/2, *p*-ERK1/2, and NOX2 in BMDMs pretreated with an ERK inhibitor, with or without IAld (*n* = 3). (I) ROS levels in these BMDMs (*n* = 3). (J–K) Phagocytosis and bacterial killing of ERK inhibitor-pretreated BMDMs with or without IAld (*n* = 3). (L) ROS levels in NAC-pretreated BMDMs with or without IAld (*n* = 3). (M–O) Effects of IAld on the phagocytic and bactericidal capacities of BMDMs with or without NAC pretreatment (*n* = 3). Data are presented as mean ± SEM. Statistical significance was determined by the unpaired two-tailed Student's t test (C-E) and one-way ANOVA with Bonferroni’s post hoc tests (G–I, K–M); *P <* *0.05, **P <* *0.01, ***P <* *0.001, ****P <* *0.0001; ns, not significant.

Protein interaction network of the 47 predicted targets (Figure 5A). Subsequent network pharmacology analysis enabled us to identify core target genes closely associated with Fc gamma R-mediated phagocytosis, including Map2k1, Ptprc, Syk, and Mapk1 (i.e., ERK2). Studies have shown that the MAPK pathway mediates oxidized low-density lipoprotein-induced NET formation through activation of NOX2-dependent ROS production.^33^ Given the crucial role of ROS in host defense against pathogens,^34-36^ this study investigated whether IAld enhances ROS production in macrophages after bacterial infection by modulating the ERK/NOX2 axis. Notably, IAld treatment significantly increased the expression levels of *p*-ERK1/2 and NOX2 proteins in BMDMs infected with *E. coli* (Figure 5B-D) and markedly elevated intracellular ROS levels (Figure 5E). To examine whether ERK activation is central to the bactericidal mechanism mediated by IAld in BMDMs, we pretreated the cells with the ERK inhibitor PD98059 (PD) to suppress ERK activation and assessed the effect of IAld on bacterial killing. The results showed that PD effectively inhibited ERK phosphorylation and ROS generation in BMDMs (Figure 5F–I). Furthermore, PD pretreatment significantly attenuated the IAld-mediated enhancement of bacterial killing (Figure 5J and K). To further validate whether the elevation of ROS levels is integral to IAld-induced macrophage bactericidal activity, we used the ROS inhibitor *N*-acetylcysteine (NAC) to suppress ROS production in BMDMs. NAC pretreatment effectively reduced ROS levels (Figure 5L) and significantly impaired the IAld-mediated bacterial killing effect compared to IAld treatment alone (Figure 5M–O). Collectively, these experimental results demonstrate that the enhancement of macrophage bactericidal capacity by IAld depends on ROS production mediated through the ERK/NOX2 axis.

### IAld enhances the bacterial-killing capacity of macrophages by promoting ROS generation via the DUSP1/ERK/NOX2 signaling axis

**Figure 6.**
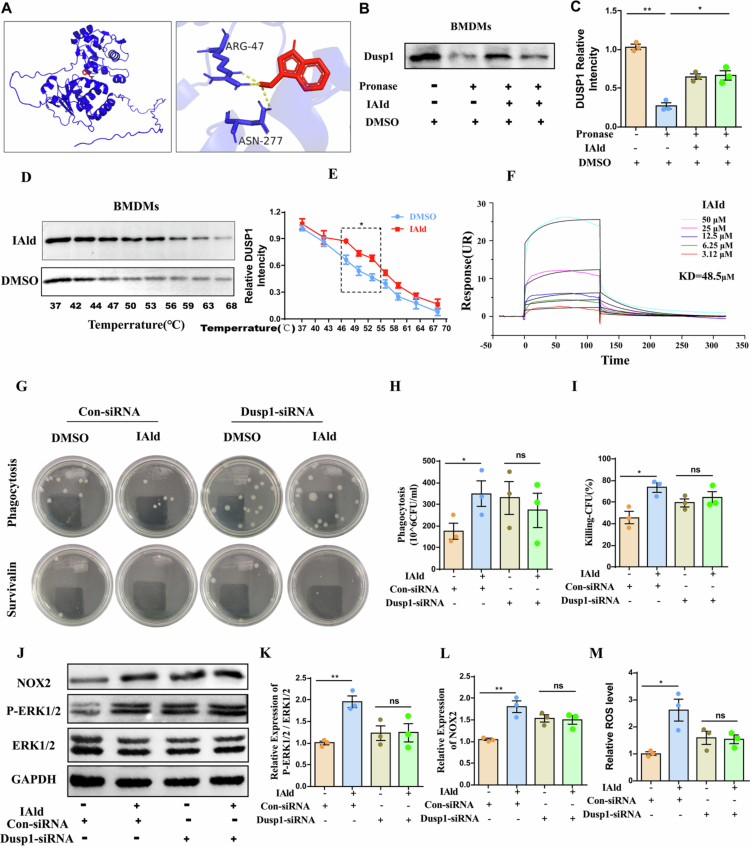
IAld enhances macrophage bactericidal capacity by promoting ROS generation via the DUSP1/ERK/NOX2 signaling axis. (A) Molecular docking analysis of the interaction between IAld and DUSP1. (B–C) Immunoblot analysis of DUSP1 in pronase-digested BMDMs lysates (*n* = 3). (D–E) Cellular thermal shift assay (CETSA) indicated DUSP1 degradation at different temperatures with or without IAld treatment (*n* = 3). (F) Surface plasmon resonance (SPR) analysis of IAld binding to DUSP1. (G–I) Phagocytosis and bacterial killing capacity of BMDMs transfected with DUSP1 siRNA or negative control siRNA for 48 hours, with or without IAld treatment (*n* = 3). (J–L) Representative Western blot images and quantitative analysis of ERK1/2, *p*-ERK1/2, and NOX2 protein expression in BMDM lysates under different treatment conditions (*n* = 3). (M) ROS levels in BMDM lysates under different treatment conditions (*n* = 3). Data are presented as mean ± SEM. Statistical significance was determined by the one-way ANOVA with Bonferroni’s post hoc tests. *P <* *0.05, **P <* *0.01, ***P <* *0.001, ****P <* *0.0001; ns, not significant.

The enhancement of macrophage bactericidal capacity by IAld is dependent on the ERK phosphorylation process. Through systematic screening of 47 common targets, we identified dual-specificity phosphatase 1 (DUSP1), a key negative regulator of the ERK signaling pathway. DUSP1 was selected for focused investigation based on the following evidence: 1) Molecular docking results revealed a stable binding mode of IAld within the active site of DUSP1. IAld specifically interacts with two key residues, ARG-47 and ASN-277, via hydrogen bonds (Figure 6A); 2) Drug affinity responsive target stability (DARTS) experiments further confirmed that IAld specifically binds to the DUSP1 protein (Figure 6B–C); 3) Cellular thermal shift assay (CETSA) results indicated that IAld significantly enhanced the thermal stability of DUSP1 in BMDMs (Figure 6D–E); 4) Surface plasmon resonance (SPR) analysis demonstrated a dose-dependent binding interaction between DUSP1 and IAld (Figure 6F); 5) IAld does not affect the expression of DUSP1 (Supplemental information FigS1I-J). Published literature reports that DUSP1 negatively regulates ERK activity through its dephosphorylation.^37^ Immunoblotting results showed that IAld treatment significantly upregulated the expression levels of phosphorylated ERK1/2 (*p*-ERK1/2) and NOX2 in BMDMs. Based on these findings, we proposed the following scientific question: Does IAld, by targeting and inhibiting DUSP1, block its dephosphorylation of ERK, thereby promoting NOX2 expression and enhancing ROS generation?

To validate whether IAld enhances phagocytic function through a DUSP1-dependent mechanism, we knocked down DUSP1 expression using siRNA. The experimental results demonstrated that DUSP1 knockdown alone promoted macrophage phagocytic function. However, DUSP1 knockdown (relative to the negative control siRNA group) significantly diminished IAld’s promotion of bacterial clearance by BMDMs after *E. coli* infection (Figure 6G–I). Furthermore, DUSP1 knockdown directly led to upregulated *p*-ERK and NOX2 expression and increased ROS generation, and IAld treatment failed to further enhance these effects (Figure 6J–M). These results indicate that IAld likely enhances macrophage phagocytic and bactericidal activity by targeting and inhibiting DUSP1, thereby blocking its dephosphorylation of ERK, which in turn promotes NOX2 expression and ROS production.

### IAld enhances the bacterial killing of human macrophage and is negatively correlated with disease severity in sepsis patients

**Figure 7.**
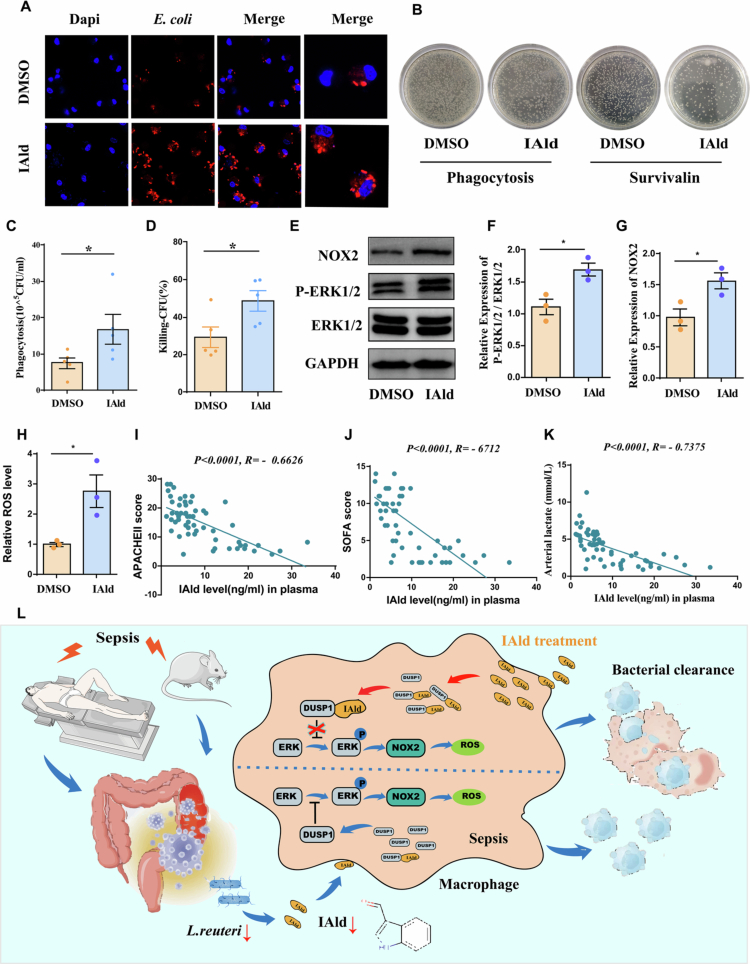
IAld enhances the bactericidal capacity of human macrophages and is negatively correlated with disease severity in sepsis patients. (A) Confocal micrographs showing phagocytosis of red fluorescent-labeled *E. coli* by MDMs pretreated with DMSO or IAld. (Scale bars: 10 μm). (B) Representative images of phagocytic activity and bacterial killing capacity in MDMs. (C–D) Quantitative results of phagocytosis and bacterial killing capacity in MDMs (*n* = 5). (E–G) Representative Western blot images and quantitative analysis of ERK1/2, *p*-ERK1/2, and NOX2 protein expression in MDMs treated with DMSO or IAld (*n* = 3). (H) IAld significantly increased ROS levels in MDMs (*n* = 3). (I–K) Scatter plots showing statistical correlations between plasma IAld levels and APACHE II score (I) SOFA score (J) and arterial lactate levels (K) in sepsis patients (*n* = 56). (L) Schematic mechanism: IAld derived from *L. reuteri* enhances macrophage bactericidal capacity by promoting ROS generation through the DUSP1/ERK/NOX2 signaling axis. Data are presented as mean ± SEM. Statistical significance was determined by Spearman rank-order correlation test (I–K) and unpaired two-tailed Student's t-test for all other comparisons. *P <* *0.05, **P <* *0.01, ***P <* *0.001, ****P <* *0.0001; ns, not significant.

Based on our foundational findings, we finally evaluated the clinical translational potential of IAld by analyzing its effects on human macrophage function. Using red fluorescent-labeled *E. coli* as phagocytic targets and confocal microscopy, we observed that IAld significantly enhanced the phagocytic capacity of monocyte-derived macrophages (MDMs) isolated from septic patients (Figure 7A). Furthermore, colony-forming unit (CFU) assays demonstrated that IAld-pretreated MDMs exhibited significantly stronger bactericidal activity against *E. coli* compared with the control group (Figure 7B–D), suggesting IAld as a potential novel compound for enhancing bacterial clearance by macrophages. Consistent with our earlier findings highlighting the essential role of ROS in IAld-induced macrophage activity, we found that IAld upregulated *p*-ERK and NOX2 expression and promoted ROS production in human MDMs (Figure 7E–H). These results were further validated using in vitro models of human macrophages. Additionally, we assessed the potential cytotoxicity of IAld (0–500 μM) on monocyte-derived macrophages (MDMs; Fig. S2D) using the CCK‑8 assay. The results showed that no significant reduction in cell viability was observed at any of the tested concentrations.

To determine whether adverse clinical outcomes correlate with reduced IAld levels, we quantified circulating IAld in patients with sepsis. These levels correlated inversely with APACHE II scores, SOFA scores, and arterial lactate levels (Figure 7I–K). Collectively, these results establish a critical protective role for IAld in human bacterial sepsis and highlight its translational promise.

## Discussion

The gut microbiota is widely recognized as a key regulator in the onset and progression of sepsis.^38^ Previous studies have confirmed that sepsis patients often exhibit an imbalance in intestinal microbial structure, characterized by a reduction in beneficial commensals and an expansion of potential pathogens.^9^ ^,^ ^39^ This dysbiosis further leads to alterations in the microbial metabolic profile, thereby influencing clinical outcomes in sepsis patients.^40^ For instance, the gut microbiota-derived metabolite hyodeoxycholic acid (HDCA) is significantly decreased in the feces of sepsis patients; supplementation with HDCA can competitively inhibit the binding of LPS to the TLR4/MD2 complex, effectively suppressing macrophage overactivation and mitigating systemic inflammation induced by sepsis.^41^ Based on such mechanisms, strategies centered on modulating the microbiota—including inhibiting pathogen expansion, restoring beneficial bacterial colonization, and supplementing beneficial metabolites—have become an important direction in sepsis intervention research.

Macrophages are key participants in the host defense against microbial infections; they recognize microbial pathogens and secrete inflammatory cytokines and chemokines to facilitate the engulfment of pathogens into phagosomes.^42^ ^,^ ^43^ However, the mechanistic interplay between alterations in the gut microbiota and the phagocytic and bacterial-killing capacities of macrophages in sepsis remains poorly understood. Therefore, this study aimed to systematically elucidate the interaction between the gut microbiota and the phagocytic and bacterial-killing capacities of macrophages in sepsis, providing a theoretical foundation for exploring potential therapeutic strategies.

Our findings align with prior research indicating that a reduction in beneficial commensals significantly affects sepsis outcomes.^28^ We observed a marked decrease in the abundance of *L. reuteri* in both sepsis patients and the CLP mouse model, and identified its protective role in CLP mice. Although previous studies have reported that *L. reuteri* can enhance intestinal barrier function and alleviate DSS-induced colitis by elevating circulating 25(OH)D levels and maintaining epithelial integrity,^38^ ^,^ ^44^ contribute to intestinal immune homeostasis,^45^ ^,^ ^46^ and ameliorate experimental autoimmune encephalomyelitis (EAE) in mice,^47^ its specific function in bacterial sepsis remained unknown. Through integrated multi-omics analysis and *in vivo*/in vitro experiments, we discovered that *L. reuteri* promotes macrophage phagocytosis of pathogens via its metabolite IAld, reduces bacterial load in CLP mice, and alleviates CLP-induced multiple organ injury. For the first time, we reveal that *L. reuteri* may serve as a gut commensal with potential for clinical application, laying a theoretical groundwork for developing sepsis adjunctive therapies based on this bacterium.

The bacterial-killing capacity of macrophages is finely regulated by reactive oxygen species (ROS) signaling,^34-36^ whose generation is driven by the MAPK signaling pathway through activation of NADPH oxidase 2 (NOX2), a process pivotal in anti-pathogen immunity.^48^ MAPK signaling activity is negatively regulated by DUSP1.^49^ Notably, emerging evidence further confirms that macrophage phagocytic and bactericidal functions are also modulated by gut microbiota-derived metabolites. For example, the microbial metabolite rhamnose can directly bind to and activate SLC12A4 in macrophages, significantly enhancing their phagocytic capacity and effectively attenuating sepsis pathology;^50^ phenylpyruvic acid (PPA) derived from Candida albicans enhances both phagocytic function and bactericidal activity of macrophages, conferring protection against sepsis;^13^ and apigenin produced by Parabacteroides goldsteinii binds to Fgr protein, thereby strengthening the phagocytic clearance capacity of macrophages. These findings collectively highlight microbial metabolites as an important source for regulating macrophage immune function.^24^

Against this background, our study focused on the regulatory role of the *L. reuteri* metabolite indole-3-carboxaldehyde (IAld) in macrophage phagocytosis during sepsis. While previous studies have shown that IAld enhances antitumor immunity by activating the aryl hydrocarbon receptor (AhR) in CD8⁺ T cells,^30^ and exhibits antiatherosclerotic^51^ and gut barrier-modulating effects,^52^ its role in regulating macrophage phagocytic clearance in sepsis remained unclear. This study is the first to demonstrate that IAld acts as a natural DUSP1 antagonist, blocking ERK1/2 dephosphorylation, increasing phosphorylated ERK1/2 (*p*-ERK1/2) levels, upregulating NOX2 expression, and thereby maintaining ROS signaling homeostasis to enhance ROS-dependent phagocytosis and bactericidal function in macrophages (Figure 7L, schematic diagram of the mechanism). This discovery offers a new perspective for sepsis therapy: the IAld-mediated immunomodulatory mechanism complements the therapeutic limitation of *β*-lactam antibiotics, which possess direct bactericidal effects but lack immunoregulatory functions. Clinical data show that circulating IAld levels in sepsis patients are significantly negatively correlated with SOFA score, APACHE II score, and lactate levels, indicating its potential not only as a protective agent but also as a clinical prognostic biomarker.

There are several limitations to this study. First, regarding the establishment of causality, although early clinical observations have shown decreased intestinal abundance of *L. reuteri* and reduced levels of IAld in septic patients, and fecal microbiota transplantation experiments have suggested a protective role, the initial 12-hour observation time point in the CLP model may be confounded by secondary effects of disease progression. Although the supplemented 6-hour data support an early declining trend, a complete causal chain requires validation through dynamic monitoring at earlier time points. Second, while we primarily focused on its effects on macrophages, its impact on other immune cells requires systematic evaluation. Mechanistically, although we confirmed that IAld mainly functions by targeting DUSP1, the involvement of other signaling pathways cannot be ruled out. Third, the CLP model used in this study did not include antibiotic intervention or source control. While this design helps to clearly dissect the host initial immune response at a mechanistic level, it differs from standard clinical practice. Moreover, in the clinical cohort, patients universally received antibiotic treatment, which may be an important confounding variable in the association between *L. reuteri* and clinical outcomes, somewhat limiting the direct translational persuasiveness of our conclusions to clinical practice. Fourth, the current intervention strategy is primarily prophylactic; however, the optimal therapeutic time window and regimen after sepsis establishment require systematic optimization. Finally, due to limitations in imaging equipment, this study could not perform *in vivo* dynamic imaging monitoring and assessment in the septic mouse model. These limitations represent challenges to be addressed in our future research.

In conclusion, this study delineates a “microbiota-immune” protective axis driven by the gut microbiota-derived metabolite IAld (from *L. reuteri*), which combats sepsis by enhancing macrophage function. Therefore, effective strategies such as treatment with IAld and similar DUSP1 inhibitors should be explored, which may provide a new approach for the clinical management of sepsis.

## Materials and methods

### Experimental animals

This investigation was conducted using male C57BL/6J wild-type mice (aged 6–8 weeks), which were obtained from the Guangdong Experimental Animal Center. All experimental procedures involving animals were reviewed and approved by the Animal Ethics and Welfare Committee (AEWC) of Guangzhou Miles Biotechnology Co., Ltd. (Approval No: MIS2023046). A severe polymicrobial sepsis model was established using cecal ligation and puncture (CLP) according to a previous study:^53^ with minor modifications. Briefly, mice were anesthetized, the cecum was exposed and ligated at approximately 66% of its length from the apex. A through-and-through puncture was then made twice with an 18-gauge needle on the antimesenteric side of the ligated cecum. A small amount of fecal content was extruded to ensure patency of the puncture site. At 12 hours post-CLP procedure, when the mice exhibited severe clinical signs of sepsis including piloerection, hunching, and reduced activity, the collection of peripheral blood, fecal samples, and tissue samples was initiated. Macrophage depletion was achieved through intraperitoneal injection of 150 µL clodronate liposomes. Transplantation of live bacteria was performed via oral gavage of 300 µL of a live *L. reuteri* (BNCC362998) suspension at a dose of 1 × 10⁹ CFU in PBS.^25^

### Human subjects

All human studies were approved by the Medical Ethics Committee of The First People's Hospital of Chenzhou (Approval No: (Scientific Research) 2023138). A total of 75 subjects were enrolled, comprising 56 sepsis patients and 19 healthy controls. The inclusion criteria for sepsis patients eligible for fecal and plasma collection were: (1) diagnosis within 24 hours of ICU admission according to Sepsis-3 criteria; (2) age between 18 and 80 y. Exclusion criteria included: (1) pregnancy or perinatal complications; (2) death within 24 hours of admission, advanced malignancy, immunocompromised status, or recent (within 3 months) use of probiotics or antibiotics; (3) missing laboratory or clinical data; (4) deemed unsuitable for participation by healthcare professionals. Plasma samples were collected after the diagnosis of sepsis but before the initiation of antibiotic therapy, while fecal specimens were obtained within 24 hours following the diagnosis of sepsis. Both plasma and fecal samples were stored at -80 °C until further analysis. The baseline characteristics of enrolled subjects are summarized in Supplementary Tables S1 and S2.

### Fecal microbiota transplantation (FMT)

Fecal microbiota transplantation was performed following established methodologies with modifications.^24^ ^,^ ^54^ For donor sample preparation, 200 mg of frozen fecal specimens collected from sepsis patients stratified by high or low *L. reuteri* abundance were homogenized in 2 mL of sterile PBS containing 25% glycerol. The mixture was sequentially filtered through multilayer membranes to obtain a homogeneous bacterial suspension. Recipient mice were acclimatized for at least one week under specific pathogen-free (SPF) conditions before initiating a 5-day antibiotic regimen (via drinking water containing penicillin, vancomycin, neomycin, and metronidazole) to deplete indigenous gut microbiota. The mice were then randomly allocated into high-*L. reuteri* and low-*L. reuteri* groups. FMT was administered once daily via oral gavage with 200  μL of fecal suspension (equivalent to 20 mg feces/mL) for five consecutive days prior to cecal ligation and puncture (CLP) surgery. Collectively, this “200 μL daily for 5 d” FMT protocol ensured effective colonization of human-derived microbiota in the intestinal tract of recipient mice.

### 16S rDNA sequencing

Fresh fecal samples were aseptically collected using sterile tubes. Genomic DNA was extracted employing the CTAB method. PCR amplification was conducted using uniquely barcoded primers, followed by purification with AMPure XT beads and quantification via Qubit fluorometry. After library preparation, the fragment size distribution and quality of the libraries were assessed using the Agilent 2100 Bioanalyzer and Kapa Illumina Library Quantification Kit, respectively. Sequencing was performed on the Illumina NovaSeq PE250 platform. During bioinformatic analysis, samples were demultiplexed according to their unique barcodes with subsequent primer trimming. Paired-end reads were merged using FLASH, and high-quality clean tags were obtained through rigorous quality filtering with fqtrim. Chimeric sequences were identified and removed using Vsearch. Denoising and amplicon sequence variant (ASV) inference were performed with DADA2, generating feature tables and representative sequences. Alpha diversity indices (including Chao1, observed species, Goods coverage, Shannon, and Simpson) were calculated using QIIME2, alongside beta diversity analysis. Taxonomic classification was conducted against the SILVA database (v138). All Statistical analyses and visualizations were implemented using the R programming language (v3.5.2).

### LC-MS/MS analysis

Metabolomic Profiling by Liquid Chromatography-Tandem Mass Spectrometry. Polar metabolite analysis was conducted following established chromatographic-mass spectrometric principles with modifications tailored to our experimental conditions.^55^ Briefly, Metabolite separation was conducted on a Thermo Scientific Vanquish UHPLC system with a Waters ACQUITY UPLC BEH Amide column (2.1 × 50 mm, 1.7 μm), coupled to an Orbitrap Exploris 120 mass spectrometer. The binary mobile phase consisted of (A) 25 mM ammonium acetate/ammonium hydroxide (pH 9.75) and (B) acetonitrile. Samples were maintained at 4 °C with 2 μL injection volume. Mass spectrometry was performed in both ionization modes under information-dependent acquisition using Xcalibur. Parameters included: sheath gas 50 arb, auxiliary gas 15 arb, capillary temperature 320 °C, spray voltages + 3.8 kV/−3.4 kV, resolutions 60,000 (full MS) and 15,000 (MS/MS), with stepped collision energies (20/30/40 eV). Raw data were converted to mzXML format and processed using XCMS-based algorithms for peak detection, alignment, and integration. Metabolite annotation was achieved by matching against BiotreeDB (v3.0), followed by multivariate analysis in SIMCA-P + 18.0.1.

### Quantitative analysis of IAld

IAld was quantified using LC-MS/MS. IAld standard and isotopic internal standard Trp-D5 were accurately weighed and dissolved in 50% methanol and pure methanol, respectively, to prepare stock solutions. These were diluted to generate mixed standard solutions at varying concentrations and a Trp-D5 working solution (4000 ng/mL). Cecal contents were homogenized with ultrapure water (1:9, g/mL). Aliquots of 300 μL cecal homogenate or 100 μL plasma were mixed with methanol (1:4, v/v) for protein precipitation. After centrifugation (13,000 rpm, 4 °C, 15 min), the supernatant was nitrogen-concentrated and reconstituted in 100 μL methanol, followed by repeat centrifugation under identical conditions. Chromatographic separation used an ACQUITY UPLC® HSS T3 column (40 °C) with mobile phase A (0.1% formic acid in water) and B (0.1% formic acid in acetonitrile) at 0.35 mL/min using an 18-min gradient. Mass detection was performed on a QTRAP® 6500 +  system in polarity switching mode with ion source temperature 550 °C, spray voltages + 5500 V/−4500 V, and optimized gas parameters. Quantification was based on specific precursor/product ion pairs with optimized declustering potentials and collision energies.

### Analysis of bacterial load in mice

Blood, peritoneal lavage fluid (PLF), and liver tissue samples were collected from mice 12 hours after CLP surgery. Serial dilutions of the samples were prepared and plated onto blood agar plates. The plates were incubated under aerobic or anaerobic conditions at 37 °C for 14–16 hours. Colony-forming units (CFUs) were counted after incubation, and the results were expressed as CFU/mL for liquid samples (blood and PLF) or CFU/g for liver tissue samples.

### Cells and cell lines

Bone marrow-derived macrophages (BMDMs) were isolated and differentiated following a previously described protocol.^56^ Briefly, bone marrow cells were collected from the femurs and tibiae of male mice and centrifuged at 1500 rpm/min, after which the supernatant was discarded. The cells were then cultured in complete DMEM medium supplemented with 1% penicillin‒streptomycin (15140‒122, Gibco) and 20 ng/mL macrophage colony-stimulating factor (M-CSF, R&D Systems). After 7 d of induction, the differentiated BMDMs were used for subsequent experiments.

Bone marrow-derived neutrophils (BMDNs) were isolated according to established protocols.^57^ Briefly, bone marrow cavities were flushed with ice-cold PBS buffer. Cells were collected by centrifugation at 427 × g for 7 minutes at 4 °C. Subsequently, Histopaque 1119 (3 mL, 11191, Sigma) and Histopaque 1077 (3 mL, 10771, Sigma) were sequentially layered into a centrifuge tube, followed by careful overlayering of the harvested bone marrow cells. Centrifugation was performed at 850 × g for 30 min at room temperature. Cells accumulating at the interface between Histopaque 1119 and 1077 were identified as neutrophils. The isolated neutrophils were collected and cultured in complete RPMI-1640 (Gibco) medium for subsequent in vitro experiments.^13^

### Phagocytosis assay

To assess the phagocytic activity of BMDMs, cells were treated with either DMSO or IAld (20–400 µM) for 2 or 24 hours, and subsequently incubated with red fluorescent-labeled *E. coli* (BNCC360097) for 45 minutes at 37 °C. After washing three times with PBS, cell nuclei were stained with antifade mounting medium containing DAPI. Images were acquired using a Leica STELLARIS confocal microscope (Leica Microsystems, Germany).

Bone marrow-derived macrophages (BMDMs) or monocyte-derived macrophages (MDMs) were pretreated with IAld (200 µM) or DMSO for 12 hours, then cocultured with E. coli at a multiplicity of infection (MOI) of 100 (100 bacteria per cell) for 45 minutes. Neutrophils were similarly pretreated with IAld or DMSO for 12 hours and then incubated with E. coli at an MOI of 100 for 45 minutes. After infection, cells were washed three times with PBS containing 50 µg/mL gentamicin, lysed with 0.5% Triton X-100, and finally plated on LB agar for 14–16 hours. Bacterial loads were quantified by manual counting of colony forming units (CFUs).

### Bacterial killing assay

Treated bone marrow-derived macrophages (BMDMs) or monocyte-derived macrophages (MDMs) were divided into two aliquots. One aliquot was infected with *E. coli* at an MOI of 100 for 45 minutes, followed by three washes with PBS containing 50 µg/mL gentamicin and lysis with 0.5% Triton X-100 to collect the immediate sample (t = 0). The other aliquot was further incubated for 60 minutes (t = 1 hour).

For treated bone marrow-derived neutrophils (BMDNs), cells were infected with *E. coli* at an MOI of 100 for 45 minutes, resuspended in medium containing 50 µg/mL gentamicin for 30 minutes, and then lysed with 0.5% Triton X-100 in PBS to assess bacterial uptake (t = 0 hours). Additional samples were incubated for another hour (t = 1 hour). The bacterial killing rate was calculated as the percentage of colony reduction using the formula: [(CFU count at 0 hour − CFU count at 1 hour)/CFU count at 0 hour] × 100%.^13^ ^,^ ^58^

### Drug affinity responsive target stability assay (DARTS)

The experiment was performed according to a previously described method.^59^ Briefly, cells were lysed with mammalian protein extraction reagent (78501, Thermo Scientific) on ice for 15‒30 minutes. After centrifugation (18,000 × g, 15 min), the protein concentration was measured and adjusted to 4 μg/μL with 1 × TNC buffer. The lysates were equally divided into 4 aliquots (each containing 400 μg total protein), followed by incubation with either specified concentrations of IAld or an equal volume of DMSO at room temperature for 2 hours. The incubated samples were then digested with pronase (4693116001, Roche) at a 1:600 (w/w) ratio for 30 minutes. Subsequently, 5× loading buffer was added and the mixtures were boiled for 10 minutes before Western blotting analysis.

### Cellular thermal shift assay(CETSA)

The experiment was conducted according to a previously established method.^60^ Bone marrow-derived macrophages (BMDMs) were collected and subjected to three freeze‒thaw cycles using liquid nitrogen. The lysates were equally divided into two aliquots and incubated with either IAld or an equal volume of DMSO at room temperature for 2 hours. Subsequently, the samples were heat-treated at graded temperatures for 3 minutes, immediately cooled at room temperature for 3 minutes, and centrifuged at 15,000 rpm for 15 minutes at 4 °C. Finally, the supernatants were mixed with 5 ×  loading buffer, boiled, and analyzed by Western blotting.

### Surface plasmon resonance (SPR) experiment

To determine the binding affinity between IAld and DUSP1, this study conducted surface plasmon resonance (SPR) experiments. A volume of 3 μL of DUSP1 at a concentration of 0.5 mg/mL was immobilized on the protein chip, followed by activation with 10 mM NiSO₄. Different concentrations of IAld (1, 5, 10, 25, and 50 μM) were injected into the mobile phase at a flow rate of 30 mm/min. The response signals were acquired and analyzed using PlexArray HT software (Plexera Bioscience, Seattle, WA, USA).

### Molecular docking analysis

The molecular weight and 3D structure of indole-3-carboxaldehyde (IAld, CAS: 487-89-8) were obtained from the PubChem database. The 3D structure of DUSP1 was downloaded from the RCSB PDB database (http://www.rcsb.org/). Using AutoDockTools software, both the ligand and protein were prepared for molecular docking. For DUSP1, its crystal structure was processed by removing water molecules, adding hydrogen atoms, modifying amino acid residues, optimizing energy, and adjusting force field parameters to obtain a low-energy conformation suitable for ligand binding. Finally, molecular docking was performed between the processed DUSP1 structure and IAld.

### Statistical analysis

The sample size for each experimental group or independent experiment is indicated in the respective figure panels. Survival curves were analyzed using the log-rank (Mantel-Cox) test. An unpaired two-tailed Student's t-test was used for determining the statistical difference between two groups, and one-way ANOVA with Bonferroni's post hoc tests was used for three or more groups. Correlation analyses were performed using Spearman's rank-order correlation test. All Statistical analyses were performed using GraphPad Prism version 10.1.2. Data are presented as mean ± SEM unless otherwise specified. A *P-*value of < 0.05 is considered significant, and the statistical sample size (*n*) is listed in each figure legend.

## Supplementary Material

Supplementary Figures.docx Supplementary Figures.docx

Supplemental information Tables.docx Supplemental information Tables.docx

## Resource availability

Lead contact: For further information and requests for resources, please contact the lead contact, Zhiwang Li, M.D. (lizhiwanghn@163.com).

Materials availability: This study did not generate new unique reagents.

Data and code availability: 16S rDNA Sequencing data have been deposited at SRA (PRJNA1337290), and is publicly available as of the date of publication.
