# Supplementary material for: Indole-3-carboxaldehyde from Limosilactobacillus reuteri targets the DUSP1/ERK/NOX2/ROS axis to enhance the bactericidal activity of macrophages and protects against sepsis
Source: Gut Microbes. 2026 May 14;18(1):2671382. doi: 10.1080/19490976.2026.2671382 (PMC13182962; doi:10.1080/19490976.2026.2671382)
Supplement: Supplementary Figures.docx [file KGMI_A_2671382_SM4728.docx]

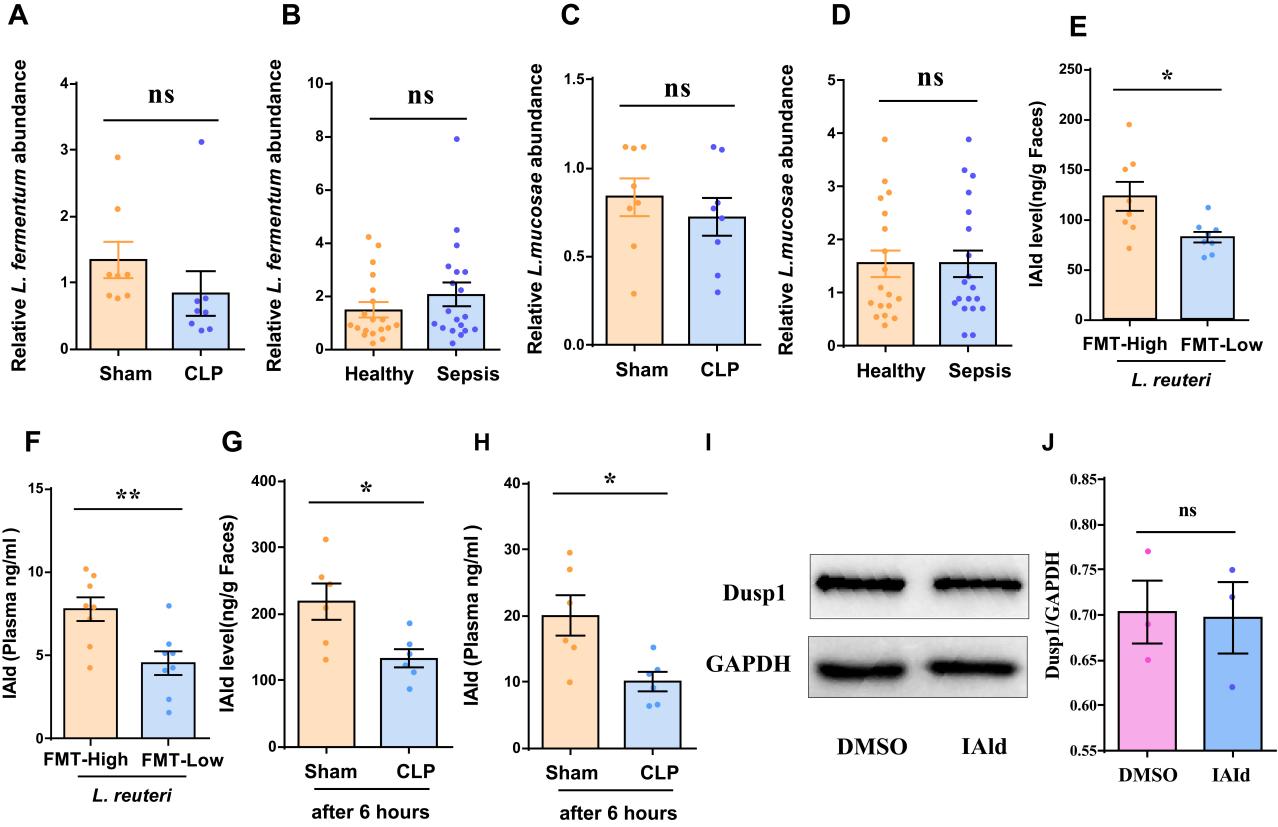


**Figure S1** (A) Quantitative PCR (qPCR) analysis of fecal *L. fermentum* abundance in sham and CLP mice (n = 8 per group). (B) qPCR analysis of fecal *L. fermentum* relative abundance in healthy controls and septic patients (n=19 per group). (C) Quantitative PCR (qPCR) analysis of fecal *L. mucosae* abundance in sham and CLP mice (n = 8 per group). (D) qPCR analysis of fecal *L. mucosae* relative abundance in healthy controls and septic patients (n=19 per group). (E) Mice receiving feces with high *L. reuteri* abundance elevated levels of IAld in feces compared to those receiving low *L. reuteri* abundance feces(n = 8 per group) . (F) Mice receiving feces with high *L. reuteri* abundance elevated levels of IAld in plasma compared to those receiving low *L. reuteri* abundance feces(n = 8 per group). (G) IAld levels in fecal of mice at 6 hours after CLP surgery(n=6). (H) IAld levels in plasma samples of mice at 6 hours after CLP surgery(n=6). (I-J)IAld does not affect the expression of DUSP1(n=3). Data are presented as mean ± SEM. Statistical significance was determined by the unpaired two-tailed Student's t test. **P < 0.05, **P < 0.01, ***P < 0.001, ****P < 0.0001*; ns, not significant.


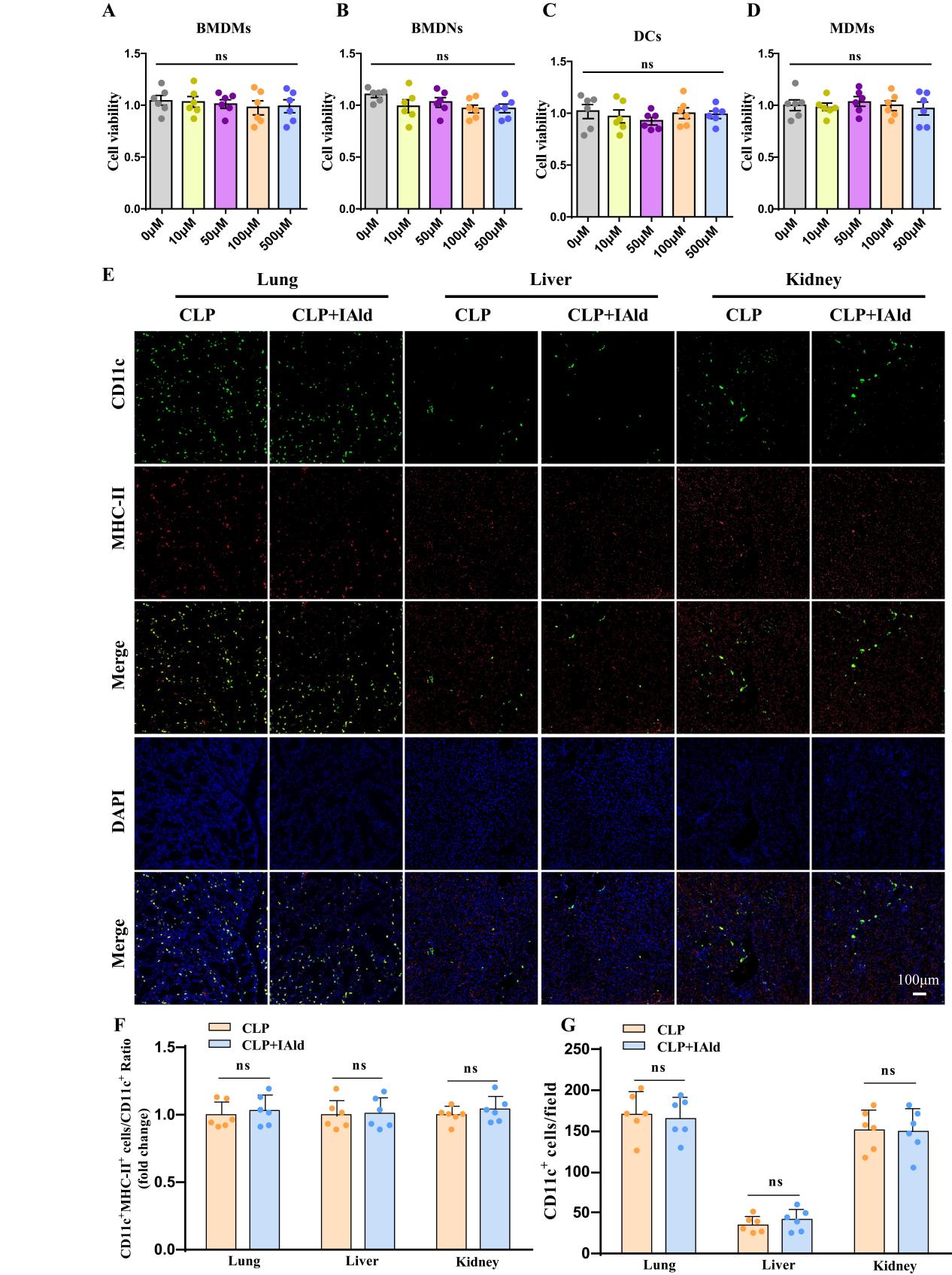


**Figure S2** (A) Within the experimental concentration range, IAld showed no significant effect on the viability of bone marrow-derived macrophages(n=6); (B) Within the experimental concentration range, IAld showed no significant effect on the viability of bone marrow-derived neutrophils(n=6); (C) Within the experimental concentration range, IAld showed no significant effect on the viability of dendritic cells(n=6); (D) Within the experimental concentration range, IAld showed no significant effect on the viability of monocyte-derived macrophages(n=6). (E) Representative immunofluorescence images showing the activation status of dendritic cells in lung, liver, and kidney tissues under IAld treatment; (F-G) Relative quantitative analysis of immunofluorescence indicating the activation level of dendritic cells in lung, liver, and kidney tissues(n=6). Data are presented as mean ± SEM. Statistical significance was determined by the one-way ANOVA with Bonferroni’s post hoc tests (A-D) and unpaired two-tailed Student's t test(F-G); **P < 0.05, **P < 0.01, ***P < 0.001, ****P < 0.0001*; ns, not significant.


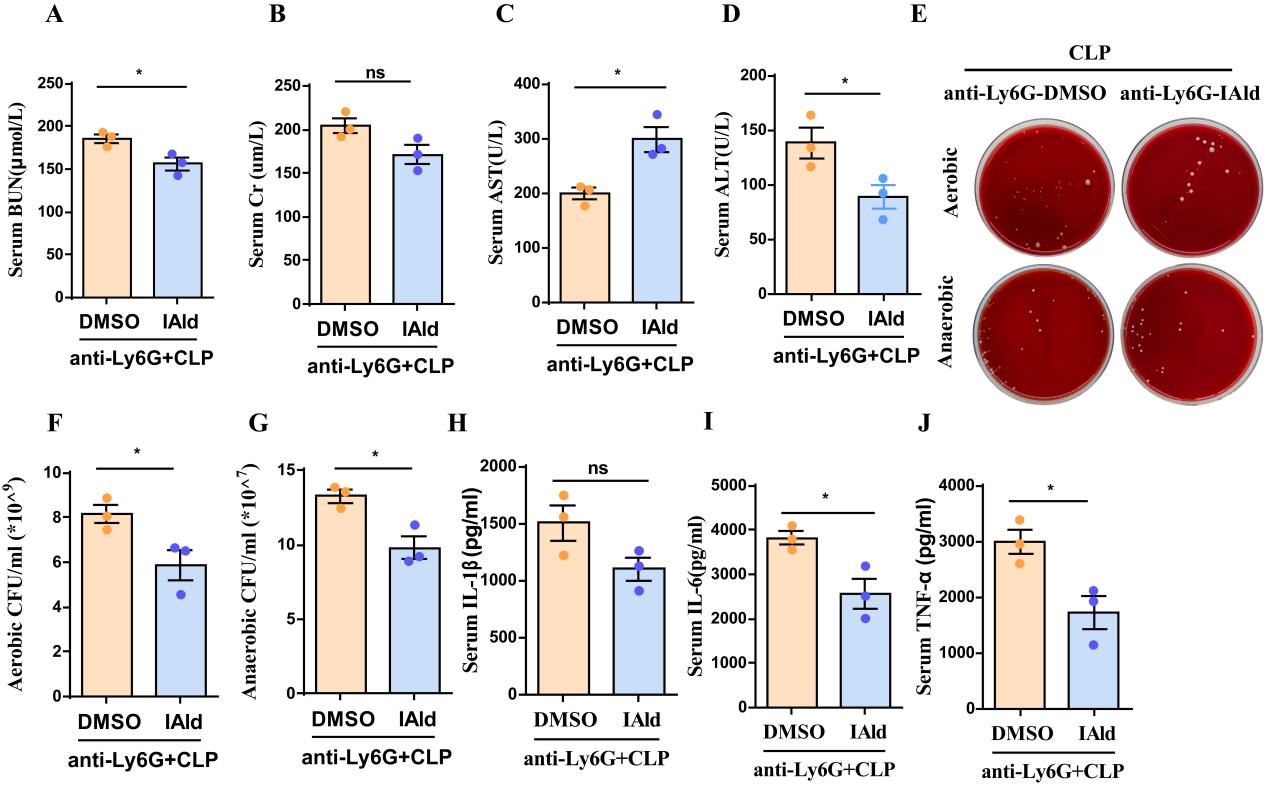


**Figure S3**

Neutrophil-depleted mice were orally pretreated with DMSO or IAld prior to CLP surgery. (A-B) Plasma ALT and AST levels (n=3). (C-D) Plasma BUN and Cr levels (n=3). (E) Representative images of bacterial load in blood. (F-G) Quantitative results of bacterial load in blood. (H-J) Plasma levels of inflammatory factors IL-1β, TNF-α, and IL-6 (n=3). Data are presented as mean ± SEM. Statistical significance was determined by the unpaired two-tailed Student's t test. **P < 0.05, **P < 0.01, ***P < 0.001, ****P < 0.0001*; ns, not significant.
