# Supplementary material for: Indole-3-carboxaldehyde from Limosilactobacillus reuteri targets the DUSP1/ERK/NOX2/ROS axis to enhance the bactericidal activity of macrophages and protects against sepsis
Source: Gut Microbes. 2026 May 14;18(1):2671382. doi: 10.1080/19490976.2026.2671382 (PMC13182962; doi:10.1080/19490976.2026.2671382)
Supplement: Supplemental information Tables.docx [file KGMI_A_2671382_SM4729.docx]

**The Gut Commensal *Limosilactobacillus reuteri* and its Metabolite Indole-3-Carboxaldehyde Confer Protection against Sepsis via the DUSP1/ERK/NOX2/ROS Axis in Macrophages**

**Table S1. Characteristics of septic patients and healthy controls related to Figure 1.**

| **Characteristics** | **Septic patients (n = 19)** | **Healthy Subjects (n = 19)** |
| --- | --- | --- |
| **Age (yr)** mean (range) ± SD | 61.21(41-81) ± 10.84 | 61.14 (41-81) ± 11.15 |
| **Sex, male (%)** | 11 (57.89) | 12 (63.15) |
| **Sepsis due to**  Peritonitis  Pneumonia  Urinary tract infections  gastrointestinal tract injuries  Skin and soft tissue infections  Parapharyngeal abscess  Other causes | 7(36.84)  2(10.53)  3(15.79)  1(5.26)  2(10.53)  1(5.26)  3(15.79) |  |
| **APACHEII score at admission**  mean (range) ± SD  Median  **SOFA score at admission**  mean (range) ± SD  Median  **Lactate level** | 17.37 (4-28)±7.85  18  8.42(2-14)±3.85  9  4.70 ± 2.84 mmol/L |  |
| **Mortality** | 2(10.52%) |  |

Abbreviations: SD, standard deviation; APACHEII, acute pathologic and chronic health evaluation II; n, denotes number of patients.

**Table S2. Characteristics of septic patients related to Figure 1,Figure 3 and Figure 7.**

| **Patient ID** | **Age** | **Gender Male(M) Female(F)** | **Diagnosis** | **Comorbidities** | **SOFA** | **APACHE II** | **Lactate level** |
| --- | --- | --- | --- | --- | --- | --- | --- |
| 1 | 59 | F | Sepsis | Urosepsis | 7 | 23 | 6.3 |
| 2 | 56 | M | Sepsis | Urosepsis | 2 | 4 | 4.2 |
| 3 | 74 | F | Sepsis | Urosepsis | 10 | 10 | 2.2 |
| 4 | 58 | M | Sepsis | Sepsis，Severe pneumonia | 11 | 22 | 5.04 |
| 5 | 68 | F | Sepsis | Gastrointestinal perforation | 4 | 5 | 1.7 |
| 6 | 62 | M | Sepsis | Adhesive intestinal obstruction | 9 | 21 | 4.2 |
| 7 | 56 | M | Sepsis | Scrub typhus (Tsutsugamushi disease) | 14 | 28 | 7.1 |
| 8 | 63 | M | Sepsis | Severe pneumonia | 6 | 13 | 2.9 |
| 9 | 65 | M | Sepsis | Enteric sepsis | 3 | 18 | 4.9 |
| 10 | 50 | F | Sepsis | Cervical cancer with lower limb infection | 2 | 6 | 0.9 |
| 11 | 81 | M | Sepsis | Gas gangrene | 12 | 12 | 1.3 |
| 12 | 78 | M | Sepsis | Gangrenous appendicitis | 10 | 26 | 5.5 |
| 13 | 67 | M | Sepsis | Hospital-acquired infection post-gastrectomy for gastric cancer | 12 | 18 | 4.32 |
| 14 | 57 | F | Sepsis | Parapharyngeal abscess | 8 | 27 | 7.2 |
| 15 | 53 | F | Sepsis | Epidemic hemorrhagic fever | 10 | 24 | 4.62 |
| 16 | 71 | F | Sepsis | Enteric sepsis | 12 | 19 | 3.22 |
| 17 | 58 | M | Sepsis | Enteric sepsis | 11 | 28 | 8.2 |
| 18 | 58 | F | Sepsis | Upper gastrointestinal perforation | 9 | 16 | 11.3 |
| 19 | 41 | M | Sepsis | Sigmoid colon perforation | 12 | 21 | 5.5 |
| 20 | 72 | M | Sepsis | Appendiceal perforation | 6 | 22 | 4.9 |
| 21 | 80 | F | Sepsis | Multiple organ dysfunction syndrome (MODS) / Multiple organ failure (MOF) | 2 | 12 | 1.37 |
| 22 | 55 | M | Sepsis | Gastric perforation | 3 | 5 | 1.5 |
| 23 | 34 | M | Sepsis | Gallstone Disease | 10 | 24 | 5.98 |
| 24 | 77 | M | Sepsis | Necrotizing fasciitis | 12 | 17 | 5.87 |
| 25 | 68 | M | Sepsis | Liver abscess with sepsis | 4 | 11 | 4.54 |
| 26 | 69 | F | Sepsis | Intra-abdominal infection | 3 | 7 | 2.89 |
| 27 | 66 | M | Sepsis | Biliary tract infection | 9 | 15 | 3.62 |
| 28 | 56 | M | Sepsis | Skin and soft tissue infection (SSTI) | 2 | 9 | 2.1 |
| 29 | 56 | F | Sepsis | Colonic mass | 2 | 6 | 1.69 |
| 30 | 49 | F | Sepsis | Gallbladder carcinoma | 4 | 8 | 2.1 |
| 31 | 40 | M | Sepsis | Urinary tract infection (UTI) | 13 | 17 | 5.63 |
| 32 | 53 | F | Sepsis | Back abscess with sepsis | 2 | 4 | 2.51 |
| 33 | 72 | M | Sepsis | diabetes mellitus | 5 | 14 | 1.23 |
| 34 | 46 | M | Sepsis | Appendiceal perforation | 3 | 6 | 3.09 |
| 35 | 67 | M | Sepsis | Necrotizing fasciitis | 11 | 17 | 2.22 |
| 36 | 73 | F | Sepsis | Urosepsis | 7 | 11 | 6.02 |
| 37 | 76 | M | Sepsis | Severe pneumonia | 5 | 13 | 4.31 |
| 38 | 53 | M | Sepsis | Gas gangrene | 2 | 8 | 1.2 |
| 39 | 64 | M | Sepsis | Enteric sepsis | 17 | 22 | 5.65 |
| 40 | 72 | M | Sepsis | Maxillofacial infection | 5 | 5 | 1.02 |
| 41 | 60 | F | Sepsis | Liver abscess with sepsis | 4 | 7 | 1.28 |
| 42 | 54 | M | Sepsis | Gastric perforation | 2 | 18 | 1.56 |
| 43 | 69 | F | Sepsis | Appendiceal perforation | 14 | 17 | 5.41 |
| 44 | 58 | M | Sepsis | Urosepsis | 2 | 7 | 1.98 |
| 45 | 56 | F | Sepsis | Enteric sepsis | 10 | 15 | 3.55 |
| 46 | 80 | F | Sepsis | Maxillofacial infection | 5 | 14 | 2.54 |
| 47 | 63 | M | Sepsis | Parapharyngeal abscess | 11 | 24 | 5.4 |
| 48 | 67 | M | Sepsis | Gangrenous appendicitis | 11 | 19 | 3.6 |
| 49 | 71 | F | Sepsis | Upper gastrointestinal perforation | 17 | 16 | 4.7 |
| 50 | 58 | F | Sepsis | diabetes mellitus | 9 | 18 | 4.09 |
| 51 | 72 | M | Sepsis | chronic renal insufficiency | 2 | 6 | 0.96 |
| 52 | 80 | F | Sepsis | Appendiceal perforation | 11 | 12 | 6.54 |
| 53 | 55 | M | Sepsis | Urosepsis | 7 | 18 | 0.97 |
| 54 | 69 | F | Sepsis | Sepsis | 2 | 22 | 2.78 |
| 55 | 66 | M | Sepsis | Enteric sepsis | 12 | 14 | 4.45 |
| 56 | 58 | F | Sepsis | Skin and soft tissue infection (SSTI) | 16 | 17 | 5.61 |

**Table S3. The primer sequences used in qPCR**

| **Gene name** | **Forward primers (5’-3’)** | **Reverse primers (5’-3’)** |
| --- | --- | --- |
| ***16S rDNA/* V3-V4** | CCTACGGGNGGCWGCAG | GACTACHVGGGTATCTAATCC |
| ***16S rDNA /*Archae** | GYGCASCAGKCGMGAAW | GGACTACHVGGGTWTCTAAT |
| ***16S rDNA/* V4** | GTGYCAGCMGCCGCGGTAA | GGACTACHVGGGTWTCTAAT |
| ***16S rDNA /*V4-V5** | GTGCCAGCMGCCGCGG | CCGTCAATTCMTTTRAGTTT |
| ***L reuteri*** | GGCGGCTGTCTGGTCTGCAA | GCTTGCGACTCGTTGTACCGTC |
| ***L. fermentum*** | GCACCTGATTGATTTTGGTCG | GTCCATTGTGGAAGATTCCC |
| ***L. mucosae*** | CACGCAGTAGCGGTGAATAC | TGACGGGCGGTGTGTACAAG |
